# Discovery of a novel accessory protein NS7a encoded by porcine deltacoronavirus

**DOI:** 10.1099/jgv.0.000690

**Published:** 2017-03-13

**Authors:** Puxian Fang, Liurong Fang, Yingying Hong, Xiaorong Liu, Nan Dong, Panpan Ma, Jing Bi, Dang Wang, Shaobo Xiao

**Affiliations:** ^1^​ State Key Laboratory of Agricultural Microbiology, College of Veterinary Medicine, Huazhong Agricultural University, Wuhan 430070, PR China; ^2^​ The Cooperative Innovation Center for Sustainable Pig Production, Wuhan 430070, PR China; ^3^​ Department of Immunology and Aetiology, College of Basic Medicine, Hubei University of Chinese Medicine, Wuhan 430065, PR China

**Keywords:** porcine deltacoronavirus, accessory protein, NS7a, subgenomic RNA

## Abstract

Porcine deltacoronavirus (PDCoV) is an emerging swine enteric coronavirus. Bioinformatics predicts that PDCoV encodes two accessory proteins (NS6 and NS7), the species-specific proteins for coronavirus. In this study, four mAbs against the predicted NS7 were prepared by using the purified recombinant NS7 protein. Indirect immunofluorescence assay demonstrated that all mAbs recognized cells transfected with an NS7 expression construct or infected with PDCoV. Western blot showed that NS7-specific mAbs recognized an additional protein band of about 12 kDa from PDCoV-infected cell lysates but not from cells with the ectopic expression of NS7. Detailed analysis suggested that this additional protein band represented a novel accessory protein, termed NS7a, a 100 amino acid polypeptide identical to the 3′ end of NS7. Moreover, NS7a is encoded by a separate subgenomic mRNA with a non-canonical transcription regulatory sequence. In summary, our results identified a third accessory protein encoded by PDCoV, which will enhance our understanding of PDCoV.

*Porcine* *deltacoronavirus* (PDCoV) belongs to the newly identified genus *Deltacoronavirus*, family *Coronaviridae*, and is an emerging enteric coronavirus [1–3]. PDCoV was originally detected in pigs in Hong Kong in 2012 [4]. However, the outbreak of PDCoV was first announced in the USA in 2014 [5–7], followed by the detection of PDCoV in Korea [8], China [9, 10] and Thailand [11], which posed a significant threat to the pig industry and gained considerable attention [12, 13]. PDCoV contains a single-stranded positive-sense RNA of approximately 25.4 kb in length. Its genome arrangement is similar to that previously described for other coronaviruses with the typical gene order of 5′UTR-ORF1a-ORF1b-S-E-M-NS6-N-NS7-3′UTR. ORF1a and ORF1b contain two-thirds of the genome and encode two viral replicase polyproteins, pp1a and pplab, which are proteolytically cleaved into 15 mature non-structural proteins [4, 14]. The last one-third of the genome encodes four structural proteins, spike (S), envelope (E), membrane (M) and nucleocapsid (N), as well as two putative accessory proteins, NS6 and NS7 [15, 16]. NS6 is predicted to be located between the M and N in the genome, while NS7 is located within the N gene in an alternative ORF and encodes a 200 amino acid peptide [5, 17]. Accessory proteins are species specific and distributed widely throughout the coronavirus genome. Previous reports indicated that accessory proteins contained in other coronavirus are non-essential for viral replication *in vitro* [18]; however, they are associated with immune modulation and viral pathogenesis *in vivo* [19–22]. The identification and extensive study of accessory proteins is essential to gain detailed insights into their function in the viral life cycle. More recently, we identified the accessory protein NS6 and its subcellular localization in PDCoV-infected cells [23]; however, no information on the putative NS7 has been reported to date.

To determine the expression of NS7 in cells infected with PDCoV and analyse its biological function in the viral replication cycle, we prepared mAbs against the putative NS7 protein. Viral genomic RNA was extracted from PDCoV-infected LLC-PK cell lysates using TRIzol Reagent (Invitrogen) and reverse transcribed using a Transcription First Strand cDNA Synthesis kit (Roche) for cDNA synthesis according to the manufacturer’s instructions. NS7 gene-specific primers (NS7-F: 5′-GCTGAATTCATGGAGTTC CGCTTAACTCCGCCAT-3′; NS7-R: 5′-CATCTCGAGCTAGAGCCATGATGCGAGGATCAG-3′) were designed based on the sequence of the PDCoV strain CHN-HN-2014 (GenBank accession number KT336560), which was isolated from piglets with severe diarrhoea on a pig farm in Henan Province, China [24]. A 603 bp fragment of the NS7 gene was amplified by reverse transcription (RT) PCR and then cloned into a pGEX-KG expression vector, named pGEX-KG-NS7. Subsequently, the recombinant plasmid was transformed into *Escherichia coli* Rosetta (DE3) and then induced with 0.8 mM IPTG. SDS-PAGE analysis showed that the recombinant fusion protein GST–NS7 (approximately 50 kDa) was expressed as inclusion bodies in *E. coli* (Fig. 1a), which were then partially purified (Fig. 1b) by supersonic schizolysis method and centrifugation as described previously [25]. The obtained inclusion bodies were treated with buffer A (50 mM Tris-base, 0.5 mM EDTA, 50 mM NaCl, 5 % propanetriol) containing 0.5 M DTT and sarkosyl, followed by renaturation with refolding buffer containing PEG4000, oxidized glutathione and reduced glutathione. The concentration of partially purified NS7 protein was 0.77 mg ml^−1^ and this was used as immunization antigen to inoculate three 6-week-old female BALB/c mice twice via subcutaneous injection at a 2 week interval. We successfully obtained four hybridoma cell lines, named 1C8, 2E11, 3G10 and 1A10, through multiple screening and subcloning. To test the specific recognition of the four mAbs to the predicted NS7 protein, a eukaryotic expression plasmid containing NS7 was constructed by cloning the NS7 gene into the pCAGGS-HA vector with primers NS7-F and NS7-R, which was then transfected into LLC-PK cells. The expression construct pCAGGS-HA-NS6 encoding NS6 [23], another accessory protein of PDCoV, was also transfected into the LLC-PK cells as control. To rule out the possibility that the mAbs react against the hemagglutinin (HA)-tag, we also constructed an expression plasmid encoding an untagged NS7 protein as control for Western blot analysis. Indirect immunofluorescence assay (IFA) was conducted with the primary antibody rabbit anti-HA mAb or the four mAbs, followed by Alexa Fluor 594-conjugated donkey anti-rabbit IgG or Alexa Fluor 488-conjugated donkey anti-mouse IgG, respectively. IFA showed that specific red and green fluorescence was observed in identical cells transfected with pCAGGS-HA-NS7, but only red fluorescence in pCAGGS-HA-NS6-transfected LLC-PK cells (Fig. 1c). Western blot assay also indicated that the four mAbs specifically recognized the expected size of HA-tagged recombinant proteins (~25 kDa) and untagged NS7 proteins in the lysates of cells transfected with pCAGGS-HA-NS7 or pCAGGS-NS7, rather than pCAGGS-HA-NS6 or empty vector pCAGGS. The expression of the fusion proteins HA-NS7 and HA-NS6 was also verified by Western blot analysis with anti-HA mAb (Fig. 1d).

**Fig. 1.**
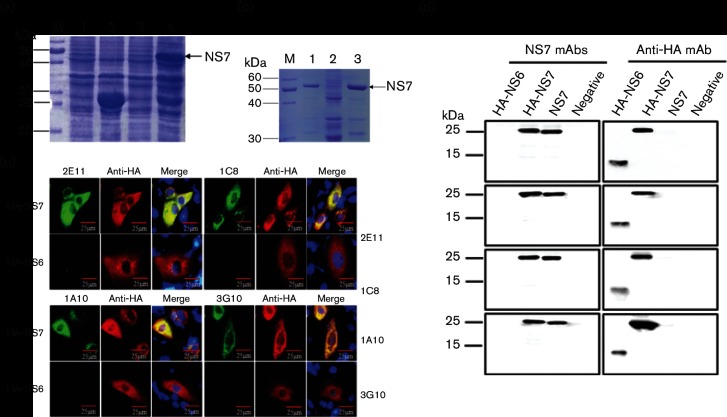
Purification of recombinant NS7 protein and generation of NS7-specific mAbs. (a) SDS-PAGE analysis of inducible NS7 expression by IPTG in *E. coli*. M, Protein molecular weight marker; lane 1, expression of empty vector pGEX-KG without IPTG induction; lane 2, inducible expression of empty vector pGEX-KG with IPTG; lane 3, expression of pGEX-KG-NS7 without IPTG induction; lane 4, inducible expression of pGEX-KG-NS7 with IPTG. (b) Representative image from SDS-PAGE analysis of purified recombinant NS7 protein. Rosetta (DE3) cells containing pGEX-KG-NS7 were induced with IPTG for 6 h and then subjected to supersonic schizolysis. Supernatant (lane 1) and precipitates (lane 2) of bacterium solution were harvested, followed by the purification of precipitates (lane 3). (c) IFA to detect the expression of NS7 in pCAGGS-HA-NS7-transfected cells. LLC-PK cells were seeded on 24-well plates followed by transfection with pCAGGS-HA-NS7 or pCAGGS-HA-NS6 as control, respectively. At 28 h post-transfection, cells were fixed and subjected to IFA with anti-HA mAb and four mAbs (1A10, 2E11, 1C8 or 3G10) against NS7, followed by the treatment with Alexa Fluor 594-conjugated donkey anti-rabbit IgG (red) and Alexa Fluor 488-conjugated donkey anti-mouse IgG (green) by confocal microscopy. Cellular nuclei were counterstained with DAPI (blue). (d) Western blotting analysis to detect NS7 in pCAGGS-HA-NS7- or pCAGGS-NS7-transfected cells with anti-HA mAb and the four mAbs described in (c). pCAGGS-HA-NS6- and empty vector pCAGGS-transfected cells act as controls, respectively.

To confirm the expression of NS7 in PDCoV-infected cells, LLC-PK cells were infected with PDCoV strain CHN-HN-2014 at a m.o.i. of 5.0. At 12 h post-infection, IFA and Western blot assay were conducted with the obtained mAbs or anti-N protein mAb [26]. As shown in Fig. 2(a), specific fluorescence was observed in cells infected with PDCoV, but no mAb recognized the uninfected cells. Interestingly, Western blotting showed that two specific protein bands were detected in PDCoV-infected LLC-PK cell lysates by each of the four mAbs. Apart from the ~25 kDa protein band, which is similar to the size observed in cells transfected with the NS7 expression construct pCAGGS-HA-NS7, a smaller protein, ~12 kDa, was also detected (Fig. 2b).

There are two possible explanations for the smaller protein band: (1) it is the degradation product of NS7 and (2) it is an additional protein encoded by PDCoV. Considering that no similar small protein band was detected in cells transfected with the NS7 expression construct pCAGGS-HA-NS7 (Fig. 1d), and at least two NS7 proteins (NS7a and NS7b) are predicted in deltacoronaviruses such as white-eye coronavirus, sparrow coronavirus and magpie robin coronavirus [4], we speculate that the observed smaller protein band may be a new unidentified PDCoV-encoded protein. To test this, we analysed whether the small protein, termed NS7a, was encoded by a separate subgenomic mRNA (sgRNA). A common feature for coronavirus is that multiple sgRNAs are generated by discontinuous transcription. Each sgRNA is composed of a short 5′ leader sequence derived from the 5′ end of viral genome and a body sequence from the 3′-poly(A) stretch to a position upstream of each genomic ORF encoding a structural or accessory protein [27, 28]. An important element in each sgRNA is the fusion site of the leader and body sequence, also termed transcription regulatory sequence (TRS) [29]. Therefore, we conducted a leader–body junction analysis to examine whether translation of the NS7a protein was initiated by a possible separate sgRNA. Intracellular total RNA was extracted from PDCoV-infected LLC-PK cells, and sgRNAs were amplified by leader–body junction RT-PCR with the primers Leader-F and NS7r (Leader-F: 5′-AATTTTATCTCCCTAGCTTCG-3′; NS7r: 5′-GAGGATCAGCCATACCCGTCTTCTC-3′). Three specific RT-PCR products (~1000, 750 and 500 bp, respectively) were obtained by agarose gel electrophoresis analysis (Fig. 3a) and then isolated from the agarose gel for cloning and sequencing. At least 10 independent clones were sequenced for each RT-PCR product. The three specific bands corresponded to sgRNA NS6, N and a new sgRNA, respectively (Fig. 3a). Sequence analysis demonstrated that the leader–body fusion site for sgRNA NS6 was ACACCT and 148 nucleotides upstream of the AUG start codon of the NS6 gene, in accord with our previous report [23]. The sgRNA N contained a leader sequence followed by the canonical PDCoV TRS (ACACCA) in accordance with predicted sgRNA N transcripts. However, the leader–body fusion site for the new sgRNA was ACCCCA, followed by 45 nucleotides and a putative ORF that was located in the C-terminal of the NS7 gene (Fig. 3b). Furthermore, there was a nucleotide difference (underlined) in the TRS sequence (ACCCCA) of the new sgRNA compared with that of the N gene (ACACCA), implying that the TRS utilized by the putative ORF is also non-canonical. In addition, the deduced protein encoded by the new sgRNA was composed of 100 amino acids and identical to the 3′ end of NS7.

It is unclear whether the new sgRNA is functional and could be used as a template for the translation of its corresponding protein. To confirm this, the putative ORF cDNA downstream from the new sgRNA (ranging from nucleotides 24 393 to 24 695) was amplified by RT-PCR with nORF-F and nORF-R (nORF-F: 5′-GCTGAATTCATGGCCCAGCTCAAGGTTTC-3′; nORF-R: 5′-TCACTCGAGCTAGAGCCATGATGCGAGGA-3′) and then cloned into a pCAGGS-HA vector. The resulting plasmid pCAGGS-HA-nORF was then transfected into the LLC-PK cells. At 28 h post-transfection, cell lysates were collected and subjected to Western blotting assays using the four mAbs against NS7. A specific protein band, ~13 kDa, was detected in the lysates of cells transfected with pCAGGS-HA-nORF, similar to the size of the NS7a protein detected in PDCoV-infected cells (Fig. 3c). To further confirm the expression of NS7a in a T7-based *in vitro* transcript system, the cDNA of NS7a was amplified with primers NS7a-F (5′-TATCTCGAGATGGCCCAGCTCAAGGTTTC-3′) and NS7a-R (5′-GCGGGTACCTTTTTTTTTTTTTTTTTTTTTTTTTTTTTTCTAGAGCCATGATGCGAGGA-3′) and cloned into the pCMVTNT vector (Promega) with *Xho*I and *Kpn*I. The resulting recombinant plasmid pCMVTNT-NS7a was linearized with *Kpn*I. The linearized DNA was transcribed by using a T7 transcription kit (mMESSAGE mMACHINE; Ambion), and the obtained total RNA transcripts were transfected into the HEK-293T cells. The RNA transcripts from empty vector pCMVTNT were used as control. At 24 h after transfection, Western blot was performed with NS7 mAb (1C8). As shown in Fig. 3(d), a specific ~12 kDa protein band could be detected in cells transfected with RNA transcripts from linearized pCMVTNT-NS7a. These results strongly suggested that the NS7a protein was translated via the new sgRNA during PDCoV infection. Because all four mAbs were generated using the full-length NS7 protein as an antigen, and they all strongly recognized NS7a, we concluded that NS7a is highly antigenic and contains the dominant epitope(s) of NS7.

As a newfound viral protein, we hope to determine whether NS7a is highly conserved in PDCoV. To date, more than 50 complete genome sequences have been determined. Using the biological software MegAlign, we analysed NS7a sequences from 30 representative PDCoV strains and found that NS7a from the CHN-HN-2014 strain was highly homologous to strains from the USA, China and Korea, with nucleotide and amino acid identities of 98.7–99.7 and 95–98 %, respectively. However, NS7a from the CHN-HN-2014 strain had a lower homology (97–98.3 and 92–94 % at the nucleotide and amino acid levels, respectively) with strains from Thailand and Lao PDR. It should be noted that strains isolated from Thailand and Lao PDR were highly similar based on the NS7a amino acid sequences. Some characteristic amino acid mutations were found in the NS7a of strains from Thailand and Lao PDR, such as mutations at A^2^ (A2V), P^59^ (P59L) and V^80^ (V80A) (Fig. S1, available in the online Supplementary Material). Of note, higher mortality rates (27.63 % in sows and 64.27 % in piglets) were reported in a pig farm infected with PDCoV in Thailand [3]. Whether these mutations in NS7a are associated with the higher mortality rate remain unclear.

Although most accessory proteins of coronavirus are not essential for viral replication *in vitro*, some accessory proteins, such as severe acute respiratory syndrome coronavirus (SARS-CoV) ORF7a [30] and ORF9b [31], are incorporated into mature virions. We also tested whether NS7 or NS7a was associated with PDCoV virions. Purified PDCoV virions were subjected to Western blot using the mAbs against NS7/NS7a, with mAb against the PDCoV N protein as a positive control. As shown in Fig. 3(e), N protein was detected in the purified virions and lysates of cells infected with PDCoV. In contrast, NS7/NS7a-specific protein bands were detected in PDCoV-infected cells but not in purified virions. These results suggested that both NS7 and NS7a are not components of PDCoV virions. Because NS7a is identical to the 3ʹ end of NS7, whether its functions are redundant remains unclear. In addition, we also used the bioinformatics softwares TMpred (http://www.ch.embnet.org/software/TMPRED_form.html) and NetOGlyc (http://www.cbs.dtu.dk/services/NetOGlyc) to analyse the potential transmembrane domain and glycosylation sites. As a result, one transmembrane domain (amino acids 69–88) and two potential *O*-glycosylation sites (S^7^ and S^12^) were found in the NS7a protein. Currently, we are generating a construct containing an infectious cDNA clone of PDCoV and hope to investigate the role of NS7a in PDCoV infection and pathogenesis.

In this study, we did not detect the sgRNA of NS7. Because the predicted TRS of NS7 is only 13 nucleotides downstream of TRS of N sgRNA, it is difficult to distinguish between NS7 sgRNA and N sgRNA using leader–body junction RT-PCR with the primers Leader-F and NS7r described in Fig. 3(a). Although other primers were tested and many clones were sequenced, the predicted NS7 sgRNA has not been determined. These observations suggest two hypotheses: (1) the abundance of NS7 sgRNA is very low and (2) the predicted separate NS7 sgRNA does not exist. However, our results clearly demonstrated that the NS7 protein was detected in cells infected with PDCoV (Figs 2b and 3c, e). A possible explanation is that the effective translation of NS7 protein is initiated by leaky scanning ribosomes from N sgRNA rather than a separate NS7 sgRNA (Fig. 3f). A similar scenario has been reported for SARS-CoV accessory protein 9b, which is encoded from a complete internal ORF within the N gene. Indeed, at least four sgRNAs (sgRNA 3, 7, 8 and 9) in SARS-CoV are bicistronic and produce two ORFs initiating at the first or additional downstream start codon [29, 31, 32]. Whether the PDCoV N sgRNA is bicistronic and the translation of NS7 is processed via leaky ribosomal scanning from N sgRNA remain to be explored.

**Fig. 2.**
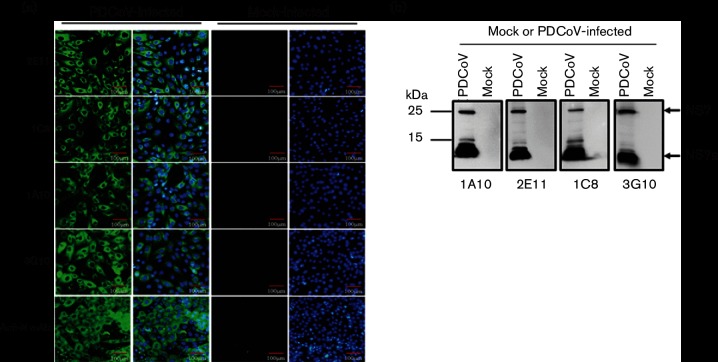
Expression of NS7 in PDCoV-infected cells. (a) IFA to detect the expression of NS7 in cells infected with PDCoV. LLC-PK cells were uninfected or infected with PDCoV at a m.o.i. of 5.0. At 12 h post-infection, the cells were subjected to IFA with four mAbs against the NS7 protein or anti-N mAb. Cellular nuclei were counterstained with DAPI (blue). (b) Western blotting analysis to detect NS7 protein. PDCoV-infected cell lysates were collected for Western blot with the four mAbs described in (a).

**Fig. 3.**
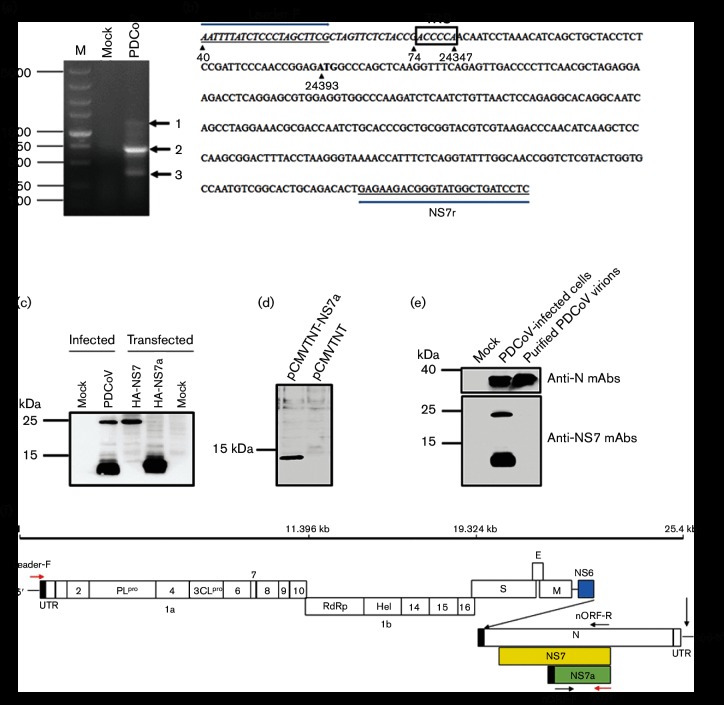
Analyses of the sgRNA and expression of NS7a. (a) A representative image from agarose gel electrophoresis of RT-PCR products amplified from PDCoV mRNA is shown. M, Molecular size ladder. The numbers ranging from 1 to 3 represent sgRNAs NS6, N and NS7a, respectively. (b) Analysis of the sgRNA NS7a sequence. The primers and the leader sequences are displayed as underlined and as italicized, respectively. The positions of the nucleotides in the genome sequences are indicated by black arrowheads. The start codon ATG in NS7a sgRNA is marked in bold. Boxed regions represent the TRS used for sgRNA synthesis. (c) Expression of NS7 and NS7a in PDCoV-infected cells or cells transfected with pCAGGS-NS7 or pCAGGS-NS7a. (d) The translation of *in vitro* NS7a transcript. The RNA transcripts from linearized pCMVTNT-NS7a or empty vector pCMVTNT were transfected into HEK-293T cells, respectively. Western blot with NS7 mAb (1C8) was performed at 24 h post-transfection. (e) The NS7/NS7a protein is not present in the purified virions. Purified virus particles were subjected to Western blot assay with mAbs against NS7/NS7a or N protein, respectively. (f) The primer design for the leader–body junction RT-PCR analysis and exact localization of NS7a are shown in a schematic diagram of the PDCoV full-length genome.

In summary, we identified the expression of NS7 protein during PDCoV infection by generating its corresponding mAbs. Furthermore, we identified the novel accessory protein NS7a encoded by PDCoV. We also confirmed the existence of a separate sgRNA corresponding to the NS7a coding region with a non-canonical leader–body fusion site. Although NS7a is conserved in PDCoV, some mutations also exist in different PDCoV isolates. Our results provide crucial information for the subsequent study of NS7a function during PDCoV infection.

## Supplementary Data

255 Supplementary File 1

## References

[1] Chen Q, Gauger P, Stafne M, Thomas J, Arruda P (2015). Pathogenicity and pathogenesis of a United States porcine deltacoronavirus cell culture isolate in 5-day-old neonatal piglets. Virology.

[2] Hu H, Jung K, Vlasova AN, Chepngeno J, Lu Z (2015). Isolation and characterization of porcine deltacoronavirus from pigs with diarrhea in the United States. J Clin Microbiol.

[3] Janetanakit T, Lumyai M, Bunpapong N, Boonyapisitsopa S, Chaiyawong S (2016). Porcine deltacoronavirus, Thailand, 2015. Emerg Infect Dis.

[4] Woo PC, Lau SK, Lam CS, Lau CC, Tsang AK (2012). Discovery of seven novel mammalian and avian coronaviruses in the genus *Deltacoronavirus* supports bat coronaviruses as the gene source of *Alphacoronavirus* and *Betacoronavirus* and avian coronaviruses as the gene source of *Gammacoronavirus* and *Deltacoronavirus*. J Virol.

[5] Li G, Chen Q, Harmon KM, Yoon KJ, Schwartz KJ (2014). Full-length genome sequence of porcine deltacoronavirus strain USA/IA/2014/8734. Genome Announc.

[6] Marthaler D, Jiang Y, Collins J, Rossow K (2014). Complete genome sequence of strain SDCV/USA/Illinois121/2014, a porcine deltacoronavirus from the United States. Genome Announc.

[7] Wang L, Byrum B, Zhang Y (2014). Detection and genetic characterization of deltacoronavirus in pigs, Ohio, USA, 2014. Emerg Infect Dis.

[8] Lee S, Lee C (2014). Complete genome characterization of Korean deltacoronavirus strain KOR/KNU14-04/2014. Genome Announc.

[9] Dong N, Fang L, Zeng S, Sun Q, Chen H (2015). Porcine deltacoronavirus in mainland China. Emerg Infect Dis.

[10] Wang YW, Yue H, Fang W, Huang YW (2015). Complete genome sequence of porcine deltacoronavirus strain CH/Sichuan/S27/2012 from mainland China. Genome Announc.

[11] Madapong A, Saeng-Chuto K, Lorsirigool A, Temeeyasen G, Srijangwad A (2016). Complete genome sequence of porcine deltacoronavirus isolated in Thailand in 2015. Genome Announc.

[12] Ma Y, Zhang Y, Liang X, Lou F, Oglesbee M (2015). Origin, evolution, and virulence of porcine deltacoronaviruses in the United States. MBio.

[13] Zhang J (2016). Porcine deltacoronavirus: overview of infection dynamics, diagnostic methods, prevalence and genetic evolution. Virus Res.

[14] Song D, Zhou X, Peng Q, Chen Y, Zhang F (2015). Newly emerged porcine deltacoronavirus associated with diarrhoea in swine in China: identification, prevalence and full-length genome sequence analysis. Transbound Emerg Dis.

[15] Chen F, Zhu Y, Wu M, Ku X, Yao L (2015). Full-length genome characterization of Chinese porcine deltacoronavirus strain CH/SXD1/2015. Genome Announc.

[16] Woo PC, Huang Y, Lau SK, Yuen KY (2010). Coronavirus genomics and bioinformatics analysis. Viruses.

[17] Lee S, Lee C (2015). Functional characterization and proteomic analysis of the nucleocapsid protein of porcine deltacoronavirus. Virus Res.

[18] Haijema BJ, Volders H, Rottier PJ (2004). Live, attenuated coronavirus vaccines through the directed deletion of group-specific genes provide protection against feline infectious peritonitis. J Virol.

[19] Curtis KM, Yount B, Baric RS (2002). Heterologous gene expression from transmissible gastroenteritis virus replicon particles. J Virol.

[20] De Haan CA, Masters PS, Shen X, Weiss S, Rottier PJ (2002). The group-specific murine coronavirus genes are not essential, but their deletion, by reverse genetics, is attenuating in the natural host. Virology.

[21] Kopecky-Bromberg SA, Martínez-Sobrido L, Frieman M, Baric RA, Palese P (2007). Severe acute respiratory syndrome coronavirus open reading frame (ORF) 3b, ORF 6, and nucleocapsid proteins function as interferon antagonists. J Virol.

[22] Siu KL, Yeung ML, Kok KH, Yuen KS, Kew C (2014). Middle East respiratory syndrome coronavirus 4a protein is a double-stranded RNA-binding protein that suppresses PACT-induced activation of RIG-I and MDA5 in the innate antiviral response. J Virol.

[23] Fang P, Fang L, Liu X, Hong Y, Wang Y (2016). Identification and subcellular localization of porcine deltacoronavirus accessory protein NS6. Virology.

[24] Dong N, Fang L, Yang H, Liu H, Du T (2016). Isolation, genomic characterization, and pathogenicity of a Chinese porcine deltacoronavirus strain CHN-HN-2014. Vet Microbiol.

[25] Liu B, Li G, Sui X, Yin J, Wang H (2009). Expression and functional analysis of porcine aminopeptidase N produced in prokaryotic expression system. J Biotechnol.

[26] Luo J, Fang L, Dong N, Fang P, Ding Z (2016). Porcine deltacoronavirus (PDCoV) infection suppresses RIG-I-mediated interferon-β production. Virology.

[27] Sawicki D, Wang T, Sawicki S (2001). The RNA structures engaged in replication and transcription of the A59 strain of mouse hepatitis virus. J Gen Virol.

[28] Sawicki SG, Sawicki DL, Siddell SG (2007). A contemporary view of coronavirus transcription. J Virol.

[29] Thiel V, Ivanov KA, Putics A, Hertzig T, Schelle B (2003). Mechanisms and enzymes involved in SARS coronavirus genome expression. J Gen Virol.

[30] Huang C, Ito N, Tseng CT, Makino S (2006). Severe acute respiratory syndrome coronavirus 7a accessory protein is a viral structural protein. J Virol.

[31] Xu K, Zheng BJ, Zeng R, Lu W, Lin YP (2009). Severe acute respiratory syndrome coronavirus accessory protein 9b is a virion-associated protein. Virology.

[32] Weiss SR, Navas-Martin S (2005). Coronavirus pathogenesis and the emerging pathogen severe acute respiratory syndrome coronavirus. Microbiol Mol Biol Rev.

