# Supplementary material for: Discovery of a novel accessory protein NS7a encoded by porcine deltacoronavirus
Source: J Gen Virol. 2017 Mar 13;98(2):173–8. doi: 10.1099/jgv.0.000690 (PMC7079566; doi:10.1099/jgv.0.000690)
Supplement: Supplementary file 1 [file jgv-98-173-s001.pdf]

Figure S1

```
CHN-HN-2014              MAQLKVSELTPSTLEEDLRSVEVAQDLNLLTPEAQAISPGNATNLHPLRYVVRPNIKLPSGLYLRVKPFLRYLATGLVLVPMSALQTLRRRVWLILASWL   100
8734/USA-IA/2014         ----------------------------------------------q-------s-r------p------------------------------------   100
CH/Sichuan/S27/2012      ----------------------------------------------q-------s--------p------------------------------------   100
CH/SXD1/2015             ---p-----------------------------------------------------------p------------fa----------------------   100
CHJXNI2/2015             -----------------------------------v----------q-------s--------p-------------a----------------------   100
CHN-AH-2004              ----------------------------------------------l----------------p-------------------v----------------   100
CHN-HB-2014              ---p-----------------------------------------------------------p------------------------------------   100
CHN-JS-2014              ----------------------------------------------q-----k-s--------p-------------a----------------------   100
HKU15-44                 ---p-----------------------------------------------------------p------------------------------------   100
HKU15-155                ---p-----------------------------------------------------------p------------------------------------   100
S5011/2015               -v--------------------------------------------l-----k-----l----p---------------a--------------------   100
0115/PDCoV/2016/Lao      -v--------------------------------h-----------l-----k-----l----p-------------a-a--------------------   100
S5015L/2015              -v--------------------------------------------l-----k-----l----p---------------a--------------------   100
TT 1115                  -v--------------------------------h-----------l-----k-----l----p-------------a-a--------------------   100
IL2768                   ----------------------------------------------q-------s--------p------------------------------------   100
Illinois121/2014         ----------------------------------------------q-------s--------p------------------------------------   100
Indiana453/2014          ----------------------------------------------q-------s--------p------------------------------------   100
Iowa136/2015             ----------------------------------------------q-------s--------p------------------------------------   100
KNU14-04                 ----------------------------------------------q-------s--------p-------------a----------------------   100
KY4813                   ----------------------------------------------q-------s--------p------------------------------------   100
MI6148                   ----------------------------------------------q-------s--------p------------------------------------   100
Michigan448/2014         ----------------------------------------------q-------s--------p------------------------------------   100
NE3579                   ----------------------------------------------q-------s--------p------------------------------------   100
NH                       ---------------------------------------------------------------p-------------a----------------------   100
NorthCarolina452/2014    ----------------------------------------------q-------s--------p------------------------------------   100
OH1987                   ----------------------------------------------q-------s--------p------------------------------------   100
OH11846                  ----------------------------------------------q-------s--------p------------------------------------   100
OhioCVM1/2014            ---p-----------------------------------------------------------p------------------------------------   100
PA3148                   ----------------------------------------------q-------s--------p------------------------------------   100
IL/2014/026PDV_P11       ----------------------------------------------q-------s--------p------------------------------------   100
SD3424                   ----------------------------------------------q-------s--------p------------------------------------   100
```

Fig.S1. Comparison of amino acid sequences of NS7a protein of different PDCoV strains. The number on the right represents the total amino acid amount of NS7a. Various PDCoV strains are listed on the left of picture, whose genbank accession numbers are as follows: Illinois121 2014 (KJ481931); HKU15-44 (NC-016990); HKU15-155 (JQ065043); CHJXNI2/2015 (KR131621); TT 1115 (KU984334); S5011/2015 (KU051641); S5015L/2015 (KU051649); IL2768 (KJ584355); KY4813 (KJ584357); MI6148 (KJ620016); NE3579 (KJ584359); OH1987 (KJ462462); OH11846 (KT381613); PA3148 (KJ584358); SD3424 (KJ584356); 8734/USA-IA/2014 (KJ769231); CHN-AH-2004 (KP757890); CHN-HB-2014 (KP757891); CHN-HN-2014 (KT336560); CHN-JS-2014 (KP757892); 0115/PDCoV/2016/Lao (KX118627); KNU14-04 (KM820765); CH/Sichuan/S27/2012 (KT266822); CH/SXD1/2015 (KT021234); NH (KU981059); Iowa136/2015 (KX022602); Indiana453/2014 (KR265851); Michigan448/2014 (KR265850); NorthCarolina452/2014 (KR265858); OhioCVM1/2014 (KJ769231); IL/2014/026PDV_P11 (KP981395).
